# Attention Deep Feature Extraction from Brain MRIs in Explainable Mode: DGXAINet

**DOI:** 10.3390/diagnostics13050859

**Published:** 2023-02-23

**Authors:** Burak Taşcı

**Affiliations:** Vocational School of Technical Sciences, Firat University, Elazig 23119, Turkey; btasci@firat.edu.tr

**Keywords:** XAI, Densenet201, GradCam, INCA, SVM, brain tumor

## Abstract

Artificial intelligence models do not provide information about exactly how the predictions are reached. This lack of transparency is a major drawback. Particularly in medical applications, interest in explainable artificial intelligence (XAI), which helps to develop methods of visualizing, explaining, and analyzing deep learning models, has increased recently. With explainable artificial intelligence, it is possible to understand whether the solutions offered by deep learning techniques are safe. This paper aims to diagnose a fatal disease such as a brain tumor faster and more accurately using XAI methods. In this study, we preferred datasets that are widely used in the literature, such as the four-class kaggle brain tumor dataset (Dataset I) and the three-class figshare brain tumor dataset (Dataset II). To extract features, a pre-trained deep learning model is chosen. DenseNet201 is used as the feature extractor in this case. The proposed automated brain tumor detection model includes five stages. First, training of brain MR images with DenseNet201, the tumor area was segmented with GradCAM. The features were extracted from DenseNet201 trained using the exemplar method. Extracted features were selected with iterative neighborhood component (INCA) feature selector. Finally, the selected features were classified using support vector machine (SVM) with 10-fold cross-validation. An accuracy of 98.65% and 99.97%, were obtained for Datasets I and II, respectively. The proposed model obtained higher performance than the state-of-the-art methods and can be used to aid radiologists in their diagnosis.

## 1. Introduction

The incidence of cancer, which is one of these diseases, is increasing day by day. Cancer prevents cells in the body from growing normally, causing damage to tissues. According to the GLOBOCAN 2020 data created by the International Agency for Research on Cancer (IARC), cancer incidence and mortality rates show approximately 19.3 million new cancer cases in 185 countries worldwide. It is estimated that these cancer cases will be 30.2 million in 2040. Brain tumors from cancer types were 308 thousand in 2020. While 168 thousand of these cases are men, 140 thousand are women. It has been reported that brain tumor cases are estimated to be around 435 thousand in 2040. In addition, according to the data collected by IARC in 2020, the number of people who died from brain tumors in the 0–85 age range is 251 thousand. It has been reported that the number of deaths in 2040 is estimated to be around 368,000 [1,2].

A brain tumor is a mass development within the brain that is created by the tissues surrounding the brain or skull and directly impacts human existence. These masses may be benign or cancerous. Brain tumors cause uneven growth inside the brain and exert pressure on the meninges. As a result of pressure, the brain experiences various problems that impair its ability to manage the body. At the onset of such symptoms as dizziness, headaches, fainting, paralysis, etc., in people, scenarios arise. Malignant tumors, as opposed to benign tumors, develop unevenly and destroy the surrounding tissues. Surgical methods often treat brain tumors. Surgery is located in a crucial region if the tumor is removed. However, medicines, radiation, etc., are preferable [3]. 

There are more than 120 different types of brain tumors in the world. Therefore, the tumor classification and grading system developed by the World Health Organization (WHO) is used today to predict the outcome of brain tumors, standardize communication, and plan treatment [4]. Generally, the cells obtained by biopsy are examined, and tumors are classified according to cell type and grade. The cell type refers to the cells that are the origin of the tumor, and nearly half of the primary brain tumors grow from glial cells. Grade refers to how the tumor cells look under the microscope and indicates aggressiveness [5]. In the WHO grading system, there is a scaling by malignancy between benign grade I and rapidly growing and difficult-to-diagnose grade IV based on identifying different histopathological groups [6,7]. In addition, depending on their origin, brain tumors are divided into primary brain tumors or metastatic brain tumors. In primary cells, the cells originate from brain tissue cells. In metastatic brain tumors, the cells become a cancerous structure in another body organ and spread to the brain [8,9].

Early diagnosis plays a crucial role in the treatment of diseases and the prevention of severe symptoms. Medical imaging techniques such as magnetic resonance imaging (MRI) [10], computed tomography (CT), single-photon emission computed tomography, Single-Photon Emission Computed Tomography (SPECT), and positron emission tomography (PET). Brain anatomy can be studied with [9]. The advantage of MRI over other medical imaging techniques is that it does not expose the patient to radiation [11]. Another significant advantage is that it offers better opportunities for imaging the anatomical structure of tissues, thanks to its ability to be unaffected by the human body. MRI is a medical method that can safely distinguish an anatomical structure from other anatomical structures by using radio waves in the strong magnetic field generated by magnets [12].

As a reflection of the rapid increase in the human population in the health sector, the amount of clinical data that needs to be handled by healthcare professionals has increased tremendously. Thus, most healthcare professionals analyze laboratory data and medical images in their daily clinical routines to investigate the presence of various diseases. Due to the increasing complexity, the increase in the specialists’ workload paves the way for the formation of critical errors in clinical decisions. Factors such as fatigue and inexperience may cause diseases to be misdiagnosed, areas without lesions to be evaluated as lesions, and especially malignant lesions to be interpreted as benign [13,14,15,16,17,18]. Le et al. [19] stated in a retrospective study that the error rate of radiological examinations was around 30%, while the daily error rate of radiologists was between 3–5%.Obermeyer et al. [20], stated that the number of diagnostic errors increased significantly, and there was not enough intervention that could reduce the errors. For this reason, computerized diagnostic systems need to be developed to assist healthcare professionals in their decisions. Explainable artificial intelligence techniques can be described as a set of techniques and methods that enable humans to interpret and understand the results of artificial intelligence. The need for explicable artificial intelligence also differs according to the target of artificial intelligence and the field in which it is applied. The need for explanation is low in a model that predicts whether an image is a cat or a dog, while the need for explanation is high in a model that predicts whether a patient has cancer. Computerized diagnostic systems are widely used to detect and diagnose breast cancer, lung cancer, prostate cancer, skin lesions, stroke, Alzheimer’s disease, and many more. Thanks to such developed systems, patterns related to diseases that experts miss in some cases can be captured with high sensitivity by computer vision techniques. Thus, it is possible to reduce healthcare professionals’ workload, use time more effectively, and increase diagnostic accuracy [21,22,23]. The development of computer diagnostic systems is significant for underdeveloped and developing countries with insufficient experts.

Studies on deep learning-based brain tumor detection in the literature are given below.

Raza et al. [24], removed the last five layers of GoogLeNet to create DeepTumorNet. Instead of these five layers, 15 new layers were added. The created hybrid CNN network was compared with nine different pre-trained models. accuracy, precision, recall, and F1 score were 99.67%, 99.6%, 100%, and 99.66%, respectively. Khazaee et al. [25], used the brats 2019 dataset to classify high-grade gliomas (HGG) and low-grade gliomas (LGG). 13,233 HGG and 13,671 LGG MR images were used in the EfficientNetB0 pre-trained model. In total, 80% of 26,904 MR images were used for training and 20% for validation. Accuracy, precision, sensitivity, and specificity were obtained at 98.87%, 98.98%, 98.86%, and 98.79%, respectively. Hamdaoui et al. [26], classified HGG and LGG using pre-trained models VGG16, VGG19, MobileNet, InceptionV3, Xception, InceptionResNetV2, DenseNet121. The Brats 2019 dataset was used as the dataset. Accuracy, precision, sensitivity, and F1-score 98.06%, 98.67%, 98.33%, and 98.62% were obtained, respectively. Tandel et al. [27] attempted to detect tumors from brain MRI images. The authors used four different datasets in their work. ResNet50, AlexNet, ResNet18, VGG16, and GoogleNet pre-trained models were used. Majority voting was applied to their predictions from these five pre-trained models. For the 1st dataset of normal and tumor classes, accuracy, sensitivity, and specificity 96.51%, 96.76%, and 96.43% were obtained with 5-fold CV, respectively. For the second dataset consisting of AST-II and AST-III classes, accuracy, sensitivity, and specificity 97.70%, 94.63%, and 99.44% 5-fold CV were obtained, respectively. For the 3rd dataset consisting of OLI-II and OLI-III classes, accuracy, sensitivity, specificity 100%, 100%, and 100% 5-fold CV results were obtained, respectively. Accuracy, sensitivity, and specificity 98.43%, 98.33%, and 98.57%, respectively, with 5-fold CV, were obtained for the 4th dataset, which consisted of HGG and LGG classes. Rizwan et al. [28] performed brain tumor classification using the Gaussian Convolutional Neural Network (GCNN). Datasets consisting of two different class T1 painful MR images were used. The classes of the first dataset are pituitary, glioma, and meningioma tumors. The classes of the second Dataset are Grade-two, Grade-three, and Grade-four. 99.80% and 97.14% accuracy were obtained for the first and second datasets, respectively. Tariciotti et al. [29] classified primary central nervous system lymphoma, glioblastoma, and solitary brain metastasis. 70% of the images in the dataset were used for training and 30% for testing. ResNet101 used the pre-trained network model. Their studies obtained an accuracy of 94.72%. Majib et al. [30], used a Kaggle dataset consisting of 253 brain MRI images. In their study, they analyzed sixteen pre-trained models. Among these pre-trained models, VGG-SCNet was used. With Stacked Classifier, F1 scores, precision, and recall were obtained at 99.20%, 99.20%, and 99.10%, respectively. Mehrotra et al. [31], classified benign and malignant tumors. Flipped image, mirrored image, noisy image, and 45° rotated preprocesses were applied to the dataset. AlexNet, GoogleNet, SqueezeNet, Resnet101 and Restnet 50 CNN networks were used. 99.04% accuracy was achieved with PT-CNN(AlexNet). Kaur et al. [32] performed brain tumor classification using Inceptionv3, InceptionResNetV2, VGG-16, Resnet101, VGG-19, Alexnet, Resnet50, GoogLeNet, and Resnet101. Three different datasets were used. 60% of the dataset was used for training and 40% for testing. Accuracy of 100%, 94% and 95.92% was achieved for the three datasets, respectively. Begum and Lakshmi [33], proposed a deep learning model for anomaly detection in brain MRI images. The method consists of 4 stages as feature extraction, feature selection, classification and segmentation stages. After the texture feature extraction process, feature reduction is performed with the oppositional gravity search algorithm, oppositional gravitational search algorithm. The reduced feature set was classified with the recursive neural network Recurrent Neural Network (RNN), and tumors in MRI images thought to contain anomalies were extracted with modified region growing algorithm. In their study, they achieved 96.26% accuracy. Saucedo et al. [34], diagnosed a brain tumor using the Grad-CAM-CNN explainable artificial intelligence model. With their proposed method, they achieved 97.11% accuracy, 95.58% sensitivity and 96.81% specificity.

Etminani et al. [35], used 3D 18F-FDG-PET images to feed the input of the 3D-CNN network. Occlusion and Grad-CAM XAI methods were used. For CN, the F1 Score was 84.00% on the 3D model, 59.00% on Resnet50 and 59.00% on InceptionV3. Kaur et al. [36], provided segmentation of medical images by combining the segmentation power of U-Net and the explainability of the Xception network with Grad-CAM features. In the 3D-IRCADb-01 dataset, 97.73% dice results were obtained.

### 1.1. Novelties and Contributions

Novel sides of this research:

- We have used deep learning as preprocessing model to create an XAI model.
- A patch-based deep feature extraction model has been proposed.
- A new XAI strategy has been used for brain tumor classification.

### 1.2. Contributions

- Brain tumor classification is a hot-topic research area for biomedical image classification, and XAI models have been proposed in this area to assist medical professionals. However, there are limited XAI models. In this research, we have proposed an XAI model to fill this gap and increase classification performance.
- To show the superiority of our proposal, we have used two public brain tumor image datasets. These datasets contain four and three categories. We obtained a pre-trained network by using a dataset with four classes and DenseNet201. Using this pretrained DenseNet201, preprocessing and feature extraction layers of our model have been created. In this respect, a deep learning-based cognitive model has been created. Moreover, our model attained superior classification performances than other state-of-art models.

## 2. Materials 

**Figure 1:**
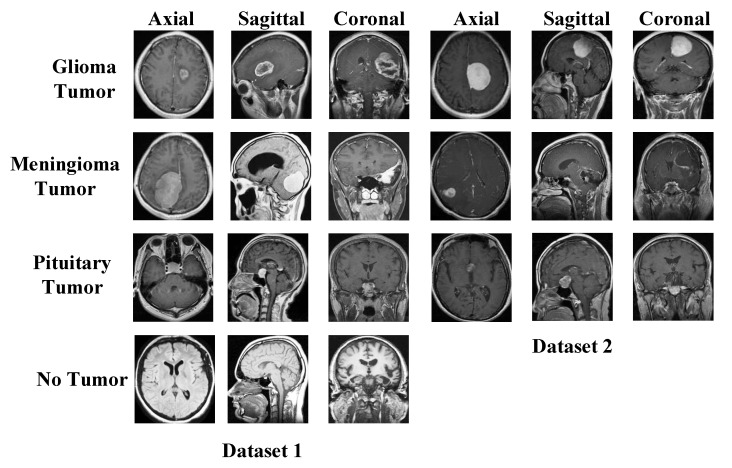
Sample images used in the two datasets. Dataset I: Kaggle dataset and this dataset have been used for training, Dataset II: Figshare dataset.

We have used two datasets in this research to show the general classification ability of the proposed model. The used both datasets are publicly available, and these are brain tumor datasets. These datasets were downloaded from Figshare and Kaggle platforms. There are three classes in the Figshare dataset, and the Kaggle dataset has four classes. Therefore, we used Kaggle Dataset for training. Sample images of these datasets are demonstrated in Figure 1.

The details of these datasets are given below.

### 2.1. Dataset I

The dataset consists of four classes of axial, sagittal, and coronal section images. The data is divided into training and test data. The glioma tumor class contains 100 images in the test folder and 826 images in the training folder. The meningioma tumor class contains 115 images in the test folder and 822 images in the training folder. There are 105 images in the test folder and 395 images in the training folder for the no-tumor class. The pituitary tumor class contains 74 images in the test folder and 827 images in the training folder. In this study, the images in the training and test folders were combined. In total, there are 926 images in the glioma tumor class, 937 in the meningioma tumor class, 500 in the no-tumor class, and 901 in the pituitary tumor class. The sum of the number of images in all classes is 3264 [37].

### 2.2. Dataset II

The dataset consists of three classes of axial, sagittal, and coronal section images. In the meningioma class, 82 patients had 708 MR images. Of the meningioma tumor class images, 209 are axial, 268 are coronal, and 231 are sagittal. There are 1426 MR images of 89 patients in the glioma tumor class. Of the glioma tumor class images, 494 are axial, 437 are coronal, and 495 are sagittal. There are 930 MR images of 62 patients in the pituitary tumor class. Of the pituitary tumor class images, 291 are axial, 319 are coronal, and 320 are sagittal. The dataset consists of 3064 T1-weighted contrast-enhanced MR images of 233 patients [38].

## 3. The Proposed DenseNet201 and Grad-Cam-Based Brain Tumor Detection Model

**Figure 2:**
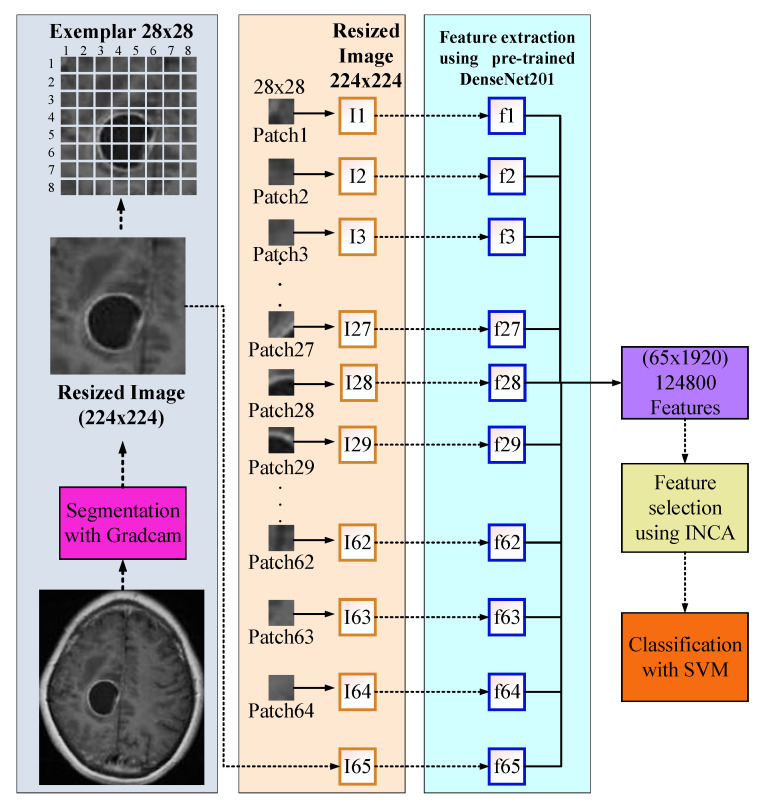
Graphical representation of the presented XAI model.

In this research, we have proposed a new explainable artificial intelligence (XAI) model for brain tumor classification. We have used two public datasets with three and four classes, respectively. Our model is a deep model, and we have used DenseNet201 for training. This model consists of 5 phases, and these phases are (i) training using DenseNet201, (ii) segmentation of tumor areas using Grad-Cam model and trained network, (iii) exemplar deep feature extraction using region of interest (ROI) and average pooling layer of the trained DenseNet201, (iv) feature selection using INCA and (v) classification with support vector machine (SVM) with 10-fold cross-validation. A graphical demonstration of the proposed XAI model is demonstrated in Figure 2.

The general steps of the proposed model are given below.

**Step 1:** Train dataset I using DenseNet201 since Dataset I has four classes.

**Step 2:** Read each image from the datasets.

**Step 3:** Apply Grad-Cam and obtain a score map. 

**Step 4:** Segment the ROI using a score map. 

**Step 5:** Resize ROI to 224 × 224.

**Step 6:** Apply patch division to the obtained ROI–segmented image–in Step 5. Herein, the size of the patch is selected as 28 × 28.

**Step 7:** Extract features from each patch using global average pooling of the trained DenseNet201 (in Step 1). 

**Step 8:** Merge the generated features.

**Step 9:** Choose the most informative features by deploying the INCA selector. 

**Step 10:** Classify the selected/chosen features using an SVM classifier with a 10-fold CV. The hyperparameters of the used SVM have been optimized using Bayesian optimization.

These ten steps have been defined in the proposed model. The steps per the phases have been given as follows. Step 1: Training, steps 2–8: patch-based deep feature extraction, Step 9: Feature selection, and Step 10: Classification. A detailed explanation of these phases is given below. 

### 3.1. Training Dataset

DenseNet201 is among the popular convolutional neural networks (CNN) in the literature. DenseNet CNN models generally consist of Dense blocks and transition layers between Dense blocks, apart from the input layer and prediction layers. The combining feature of dense blocks is formulated as given in equation 1, where x is the output feature maps, H is the layer, and i is the current number of layers. Because each layer is composed of dense blocks, the properties of all the layers that came before it is reused. Consequently, the problem of vanishing gradients is alleviated, and feature propagation is strengthened. In addition, the utilization of a limited number of filters brought about a reduction in the total number of parameters utilized by the model. Dense blocks consist of consecutive batch normalization, ReLU, and convolution operations. While the size of the feature maps remains constant within dense blocks, the number of filters varies between blocks (3 × 3–1 × 1). Between dense blocks, there are layers called transition layer, which includes 1 × 1 convolution and 2 × 2 average pooling operations. The growth rate is a hyperparameter that regulates how much information is added to the network at each layer. Concatenate is performed for this operation. DenseNet201 size is 80 MB. The number of parameters is approximately 20 million. The layer depth is 708. The image size used in the network login is 224 × 224 [39].

(1) $$x_{i}=H_{i}\left(\left[x_{0},x_{1},\ldots x_{i-1}\right]\right)$$

This work requires a pre-trained network for creating a deep feature engineering model. Therefore, DenseNet201 has created a pre-trained model using brain tumor images. The pre-trained networks have generally trained on ImageNet1k, but ImageNet1k is not related to MR images. Therefore, we used Dataset 1 to obtain a pre-trained network. The parameters used for training are given below (Table 1). The elapsed time for the training to finish is 476 min and 57 s.

**Figure 3:**
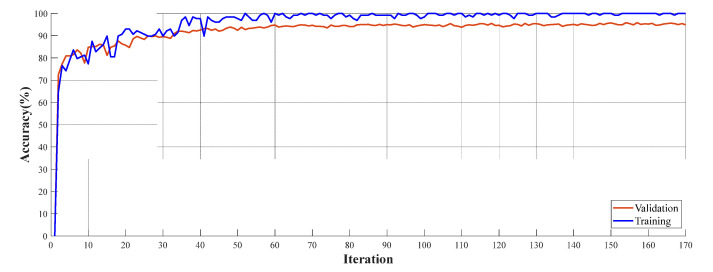

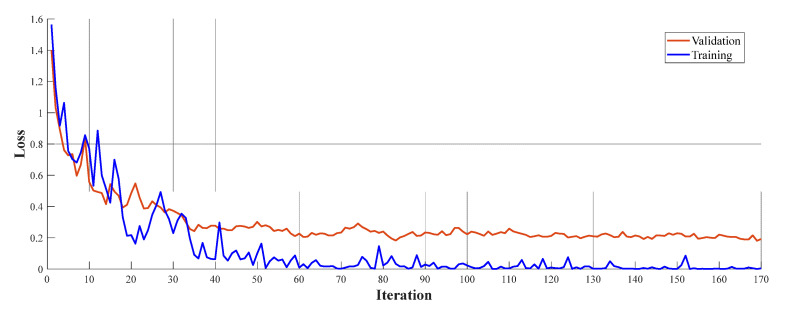
Training and validation curves of the DenseNet201 for Dataset I.

Accuracy and loss curves for training and validation are demonstrated in Figure 3.

### 3.2. Preprocessing 

**Figure 4:**
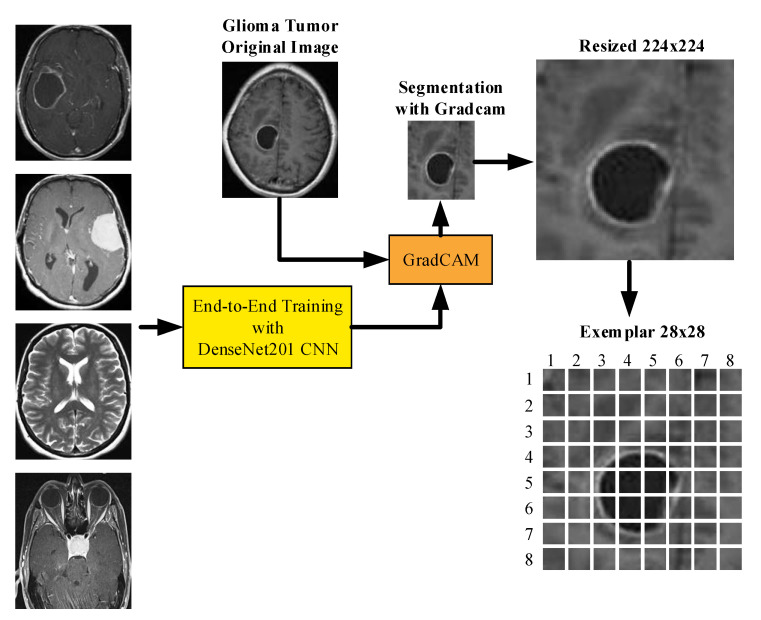
Illustration of preprocessing.

The explainable artificial intelligence (XAI) statement supports not only the function of the algorithm in producing output but also in communicating to the user how the system obtains a certain result. Recently, Gradient Weighted Class Activation Mapping (Grad-CAM) has been used to provide visual explanation and interpretability of artificial intelligence predictions [40]. The score map preprocess obtained with Grad-Cam is shown in Figure 4.

Grad-CAM determines the difference between a differentiable output, such as a class score, and the convolutional features in the chosen layer [41]. The neuron weights are found by adding the gradients over space and time. After that, these weights are put to use to combine the activation maps linearly and determine which features are most significant when it comes to producing a forecast. Assume you have a 2-D image classification network with output $o^{c}$, indicating the class c score, and you wish to compute the Grad-CAM map for a convolutional layer with k feature mappings (channels), $B_{i,j}^{k}$ where i,j indices the pixels [42].

(2) $$b_{k}^{c}=\frac{1}{N}\underset{i}{\sum }\underset{j}{\sum }\frac{\partial o^{c}}{\partial \;B_{i,j}^{k}}$$

The neuron weight is where N represents the total number of pixels in the feature map. The Grad-CAM map is, after that, a weighted mixture of the feature maps with a ReLU:

(3) $$M=RELU\left(\sum_{k}b_{k}^{c}B^{k}\right)$$

The ReLU activation ensures that you only receive features that provide value to the class of interest. As a result, the output is a heat map with the same dimensions as the feature map for the selected class. The Grad-CAM map is then upsampled to the size of the input data.

**Figure 5:**
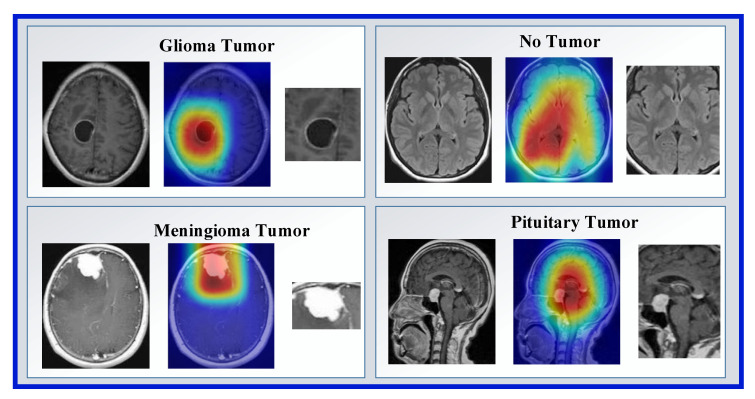
Examples of ROI and hot map images.

In this phase, our main objective is to detect tumor areas from MR images. We have used Grad-Cam to segment ROI. In the first step of this phase, we read each image from Dataset 1 and Dataset 2. The pre-trained DenseNet201 with Dataset 1 and Grad-Cam has generated a score map (hot map). By using hot areas, the ROI has been obtained. Examples of ROI and hot map images obtained with Grad-Cam are shown in Figure 5.

### 3.3. Feature Extraction

A deep feature extraction model has been presented in this work. Patch-based deep feature extraction has been used to provide a high classification ability of the patch-based model in our model. To create a feature engineering model, we need a feature extraction function. We have used a deep feature extraction function, and this deep feature extraction function is the created pretrained DenseNet201. We have used the global average pooling layer to extract features; by using this layer, 1920 features have been extracted from a patch. The steps of our proposed feature extraction model have been listed below.

1: Resize ROIs to 224 × 224 sized images.

2: Apply the fixed-size patch division and obtain 64 patches from an ROI. Herein, 28 × 28 sized patches have been used. We tested patches with variable sizes, and the best accurate patch size is obtained as 28 × 28. 

3: Extract deep features from ROI. This feature vector is the first feature vector with a length of 1920.

4: Extract deep features from each patch. By using patched, 64 more feature vectors with lengths of 1920 have been generated.

5: Merge 65 feature vectors generated to create the final feature vector with a length of 124,800 (=1920 × 65). 

### 3.4. Feature Selection

The k-NN technique was the foundation for developing a non-parametric and embedded method known as the Neighboring Component Analysis (NCA). The NCA algorithm’s primary objective is to learn the feature weighing vector by optimizing the classification accuracy with an optimum editing parameter. This is accomplished through the learning process. NCA provides information on essential qualities in addition to ranking those features is one of the advantages of using NCA [43]. NCA is a particularly useful feature selection model among the many different feature selection methods. However, it cannot calculate the optimum number of features to include. Because of this, a variant of neighborhood component analysis (NCA) called iterative neighborhood component analysis (INCA) [44] was utilized. This is the form of NCA that can count the number of features. INCA can perform an iterative feature selection procedure in conjunction with an error calculator, which enables the automatic selection of the optimum number of features. To attain the best possible degree of accuracy in classification, these feature generation and selection algorithms aim first to generate functional characteristics and then pick the most distinguishable ones. After analyzing the accumulated loss data, the smallest error values and most advantageous characteristics are chosen. An interval for iteration has been set to cut down on the amount of time required by INCA, an iterative selector with high temporal complexity. INCA was utilized as the error calculator in this investigation, with the lower and higher bounds of the classifier and iteration set to 100 and 900, respectively. kNN had the following properties: k-value: 1, distance metric: Euclidean, voting: None, and k-fold cross-validation: 10.

### 3.5. Classification

Bayesian optimization is used in this study to adjust the parameters of the SVM classifier. Bayesian optimization (BO) is a sequential experiment design method for the global optimization of functions with unknown input-to-output relationships. BO uses a sequential optimization process that iteratively decides which new data points to evaluate based on the given inputs and updates the model of the optimized objective function. BO can find the optimum value with fewer experiments compared to conventional experimental design strategies. For this reason, it is often used to optimize functions that are expensive to evaluate [45]. Considering the cost and time of experiments with these parameters, it is important to reach the optimum formulation with fewer experiments.

The goal function for Bayesian optimization was developed by employing the training dataset as well as the validation dataset as inputs. The validation dataset’s classification error is the value that is returned by the objective function after a convolutional neural network has been trained. Because Bayesian optimization chooses the optimum model based on the error rate in the validation dataset, over-learning likely occurred in the final mesh when applied to the validation dataset. To circumvent this problem, the final model under consideration is put through its paces on an independent test dataset to determine the generalization error.

The objective function performs the following steps:

- As input for optimization, variable class values are used. The objective function is defined in the objective function with the valid values of the optimization variables in a table where each column name matches the variable name.
- Training options for network architecture and optimization are defined.
- The network is trained and validated.
- The trained network is saved with training options on validation error and optimization.

**Figure 6:**
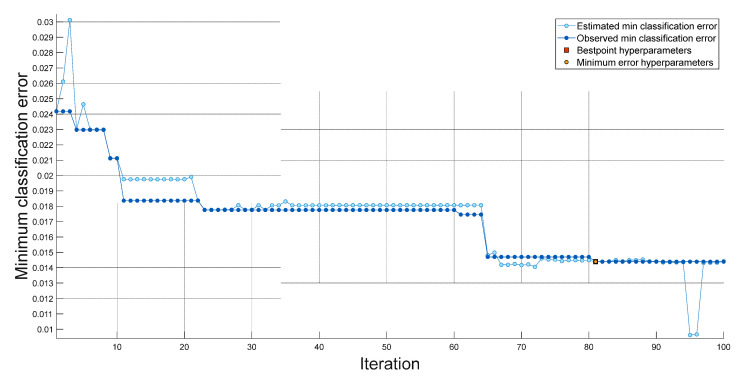
Graph of classification errors versus iterations obtained using SVM for dataset 1.

**Figure 7:**
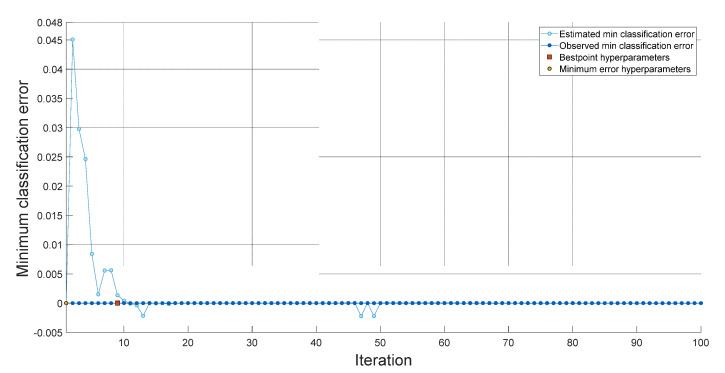
Graph of classification errors versus iterations obtained using SVM for dataset 2.

Within the scope of this research, we implemented the INCA, SVM, and bayesian optimization methodologies to develop a novel feature engineering model. We achieved good classification performance by utilizing a shallow SVM classifier in conjunction with our feature engineering model. The hyperparameters of the fine-tuned SVM obtained with 100 iterations for a dataset I are shown in Figure 6. The hyperparameters of the fine-tuned SVM obtained with 100 iterations for Dataset II are shown in Figure 7. The hyperparameters used for datasets I and II are tabulated in Table 2.

Best point hyperparameters for datasets I and II were calculated using bayesian optimization. Best point hyperparameters had 98.6% accuracy with SVM for Dataset I and 100% accuracy with SVM for Dataset II.

## 4. Experimental Results

The proposed XAI-based brain tumor classification model is programmed using MATLAB2021 on a personal computer with 64 GB of memory, an Intel i9-11900 processor, and a Windows 10 operating system without running parallel operations or needing graphics or tensor processing units. Standard performance metrics used include F1-score, specificity, precision, accuracy, and recall. 

**Figure 8:**
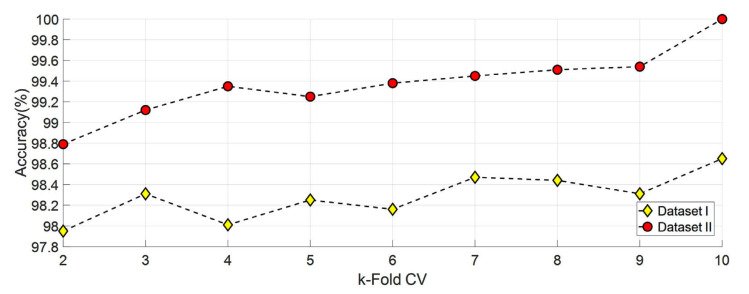
Accuracy graph obtained using SVM classifier for various k-fold CVs.

**Figure 9:**
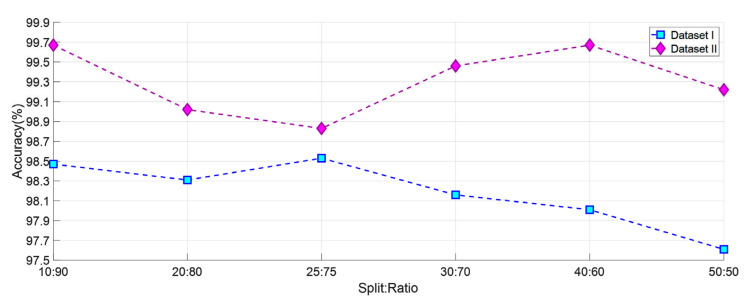
Accuracy graph obtained using SVM for various split ratios.

Different options were tested to decide which split: ratio or k-fold CV to use. First, classification is completed using SVM for datasets I and II from 2-fold CV to 10-fold CV. Obtained results are shown in Figure 8. In addition, datasets I and II were classified using SVM using six different split: ratio ratios In the literature, studies were carried out using different split ratio ratios [46,47,48,49,50,51,52,53,54,55]. The accuracy values obtained with the split ratio ratios of 10:90, 20:80, 25:75, 30:70, 40:60, and 50:50 are shown in Figure 9. As a result, ten- fold CV for Dataset I and dataset II has been chosen in this study because it gives the highest accuracy value.

**Figure 10:**
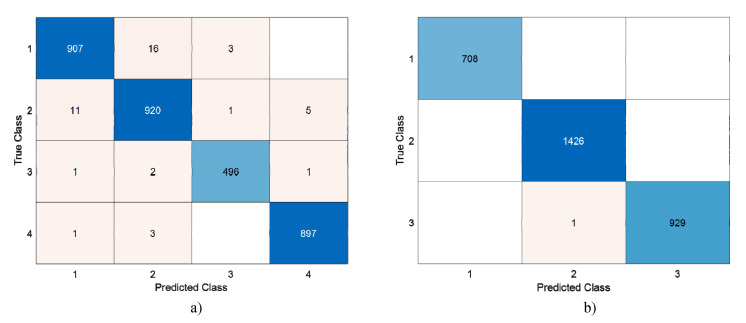
Confusion matrices obtained using SVM with 10-fold CV. Legend: (**a**) Dataset I 1 = Glioma Tumor, 2 = Meningioma Tumor, 3 = No Tumor, 4 = Pituitary Tumor, CV (**b**) Dataset II Legend 1 = Meningioma Tumor, 2 = Glioma Tumor, 3 = Pituitary Tumor.

124,800 features were obtained from DenseNet201 with the help of 28 × 28 patches. These features are selected with the help of INCA. With INCA, 231 features were selected for Dataset I, and 220 features were selected for Dataset II. The selected features were classified by running 100 times with SVM 10 fold CV. Confusion matrices of the highest accuracy achieved are shown in Figure 10.

Table 3 shows the accuracy values of different patch sizes and classifiers with 10-fold CV. The SVM classifier was chosen because it was more successful than the KNN, tree, and ANN classifiers. In addition, the highest accuracy values were obtained with the patch size 28 × 28 SVM classifier and 10-fold CV.

In the proposed method, the features obtained with INCA were classified by running 100 times with SVM 10-fold CV. TP, TN, FN, and FP values were obtained from the confusion matrix obtained. Accuracy, precision, recall, and F1-Score values were calculated with the results obtained 100 times. Calculated values (mean ± sd) are tabulated in Table 4.

**Figure 11:**
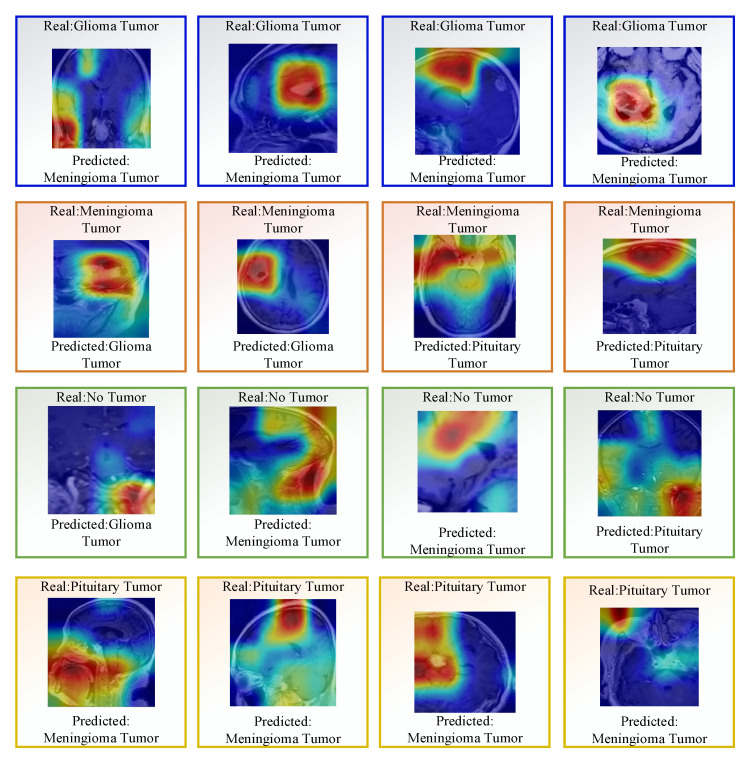
Few misclassified images using Densenet201.

In addition, some misclassified images for dataset I are shown in Figure 11.

## 5. Discussion

Detection or classification of brain tumors is crucial in saving patients’ lives. Therefore, in this study, we presented the INCA feature selective-based SVM classification model using brain MR images. This model is a CNN-based decision support system with explainable artificial intelligence (XAI). Two datasets with four classes and three classes were used. Dataset I DenseNet201 with four classes was trained with a CNN network. This trained network was used to segment the MR images using Grand-Cam datasets and obtain features. The 124,800 features obtained were selected with INCA. Finally, an accuracy of 98.65% for Dataset I and 99.97% for Dataset II were obtained using SVM classifier with 10-fold cross-validation. The summary of comparison with the state-of-the-art techniques is shown in Table 5.

As can be seen in Table 5, brain tumor detection datasets with three and four classes were used. The proposed method was applied for the Kaggle brain tumor dataset I, which consists of four classes with 3264 images, and provided 98.65% accuracy. In addition, the proposed method was applied to the figshare brain tumor dataset I, which consists of three classes with 3064 images, and provided 99.97% accuracy. Datasets I and II, widely used in the literature, were used to compare the success of the proposed method. Basaran et al. [38] used a traditional machine learning which is a time-consuming and tedious task. Authors in [56] used CNN architectures and shallower mesh. In [57,62,63,65,68,70], a 5-fold CV was used, and the results obtained with their method were lower than the proposed model. Different split: ratio ratios were used in [58,61,66,67,69,71]. The results obtained are lower than the results of our proposed model. Although 10-fold CV was used as in the method in [64], the accuracy obtained with Inception-v3 was lower.

## 6. Conclusions

In this study, we aimed to classify brain tumors using XAI. DenseNet201 architecture. End-to-end training was performed using the DenseNet201 architecture. This trained architecture was used to segment the MR images using Grand-Cam datasets and obtain features. The segmented images were resized to 224 × 224. Then the images were divided into 28 × 28 patches. The attributes of the split patches were extracted using DenseNet201 trained with Dataset I. Extracted features were selected with the INCA feature selector and classified using SVM with a 10-fold CV. The proposed method obtained 98.65% accuracy for Dataset I and 99.97% for Dataset II. To support the physicians in diagnosing, XAI has been employed to discover unknown information from medical images. The solution we developed based on datasets labeled by publicly available experts provided positive results regarding the brain tumor classification targeted in this study and outperformed existing methods in delivering superior results.

In the future, we plan to conduct a multidisciplinary study with specialist doctors and make comparative studies using SHAP, LIME, and GradCam tools on new data sets. In addition, XAI can be integrated into different diagnostic applications (Eye, Skin lesions, other cancer types, etc.).

## Figures and Tables

**Table 1:** Training Parameters.

Solver		Basic		Sequence		Advanced	
Solver	sgdm	Validation Frequency	1	Sequence Length	Longest	L2 Regularization	0.0001
Initial Learn Rate	0.01	Max Epochs	20	Sequence Padding Value	0	Gradient Threshold Method	I2norm
Iteration	170	Mini Batch Size	128	Sequence PaddingDirection	right	Shuffle	every Epoch

**Table 2:** Summary of hyperparameters used for datasets I and II.

Hyperparameters	Dataset I	Dataset II
**Multiclass method**	One-vs-All	One-vs-One
**Box Constraint level**	616.6747	987.8273
**Kernel Scale**	2.2347	-
**Kernel Function**	Gaussian	Linear
**Standardize data**	False	False

**Table 3:** Classification performance obtained for the proposed method using various classifiers.

Datasets	Patch Size	SVM (%)	Cubic KNN (%)	Fine Tree (%)	ANN (%)
**Dataset I**	28 × 28	**98.65**	96.30	91.30	97.60
	56 × 56	97.60	96.00	86.30	96.00
	112 × 112	96.70	95.40	85.90	95.60
**Dataset II**	28 × 28	99.97	96.10	99.93	98.50
	56 × 56	**99.93**	95.00	95.60	96.50
	112 × 112	96.50	95.70	85.30	95.60

**Table 4:** Classification metric calculations based on proposed feature extraction and INCA feature selection (%).

Datasets	Accuracy (% ± sd)	Precision (% ± sd)	Recall (% ± sd)	F1-Score (% ± sd)
**Dataset I Glioma Tumor**	98.42 ± 0.11	97.99 ± 0.17	97.59 ± 0.19	98.40 ± 0.26
**Dataset I Meningioma Tumor**		97.63 ± 0.18	97.99 ± 0.23	97.28 ± 0.23
**Dataset I No Tumor**		99.08 ± 0.12	99.06 ± 0.22	99.10 ± 0.12
**Dataset I Pituitary Tumor**		99.30 ± 0.09	99.36 ± 0.16	99.24 ± 0.11
**Dataset II Meningioma Tumor**	99.96 ± 0.01	100 ± 0.00	100 ± 0.00	100 ± 0.00
**Dataset II Glioma Tumor**		99.92 ± 0.02	100 ± 0.00	99.96 ± 0.01
**Dataset II Pituitary Tumor**		100 ± 0.00	99.98 ± 0.03	99.94 ± 0.02

**Table 5:** Comparison of results with the state-of-the-art techniques.

Ref. (Year)	Dataset	Model	Split: Ratio	Results (%)
Alanazi et al. (2022) [56]	Dataset I Dataset II	22 Layer CNN	80:20	**Dataset I** Accuracy = 95.75 Precision = 95.15 Sensitivity = 96.32 **Dataset II** Accuracy = 96.90
Basaran et al. (2022) [57]	Dataset I	Gray level co-occurrence matrix (GLCM), Local Binary Pattern (LBP), AlexNet, VG16, EfficientNet, ResNet, PSO, GA, ABC, SVM	5 Fold CV	Accuracy = 98.22 Precision = 97.84 Sensitivity = 98.27 Specificity = 99.43 F1 Score = 98.04
Belciug et al. (2022) [58]	Dataset I	DE/CNN	Train:2870 image Test:394 image	Accuracy = 90.04
Amou et al. (2022) [59]	Dataset II	Modified VGG16 CNN	90:10	Accuracy = 98.70 Precision = 98.30 Sensitivity = 98.60 F1 Score = 98.60
Asthana et al. (2022) [60]	Dataset II	Hanman–Renyi transform, HSTC Classifier	5 Fold CV	Accuracy = 98.91 Sensitivity = 98.92 Specificity = 99.47
Rasool et al. (2022) [61]	Dataset II	Google-Net + SVM	80:20	Accuracy = 98.10 Precision = 98.20 Recall = 98.10
Kumar et al. (2021) [62]	Dataset II	ResNet-50 CNN with global average pooling added	5 Fold CV	Accuracy = 97.08 Precision = 98.3 Sensitivity = 98.6 F1 Score = 98.6
Diaz-Pernaz et al. (2021) [63]	Dataset II	Multi-pathway CNN	5 Fold CV	Accuracy = 97.30
Noreen et al. (2021) [64]	Dataset II	Transfer learning with Inception-v3 Ensemble2 model	10 Fold CV	Accuracy = 93.79
Kakarla et al. (2021) [65]	Dataset II	Average pooling convolutional neural network (AP-CNN) model	5 Fold CV	Accuracy = 97.42 Precision = 97.41 Recall = 97.42
Alhassan et al. (2021) [66]	Dataset II	Hard swish-based RELU activation function-CNN	80:20	Accuracy = 98.60 Precision = 99.60 Recall = 98.60 F1 Score = 99.00
Gab et al. (2021) [67]	Dataset II	PGGAN-augmentation VGG19 + Bi-GRU	Training:70 Test:15 Validation:15	Accuracy = 98.54 Precision = 97.69 Sensitivity = 97.69 Specificity = 98.93 F1 Score = 97.69
Gu et al.(2021) [68]	Dataset II	Custom CNN model (Convolutional dictionary learning with local constraint (CDLLC)	5 Fold CV	Accuracy = 96.39 Precision = 94.61 Sensitivity = 94.64 F1 Score = 94.70
Noreen et al.(2020) [69]	Dataset II	Transfer learning with Inception-v3, Densenet201	80:20	Inception-v3 Accuracy = 99.34 Densenet201 Accuracy = 99.51
Ghassemi et al.(2020) [70]	Dataset II	DCGAN CNN-GAN	5 Fold CV	Accuracy = 95.60 Precision = 95.29 Sensitivity = 94.91 Specificity = 97.69 F1 Score = 95.10
Ismael et al.(2020) [71]	Dataset II	ResNet50 CNN model	80:20	Accuracy = 99.00 Precision = 99.00 Recall = 99.00 F1 Score = 99.00
Proposed Model	Dataset I Dataset II	Exemplar, DenseNet201, GradCam,SVM	10 Fold CV	**Dataset I** Accuracy = 98.65 **Pituitary** Precision = 99.34 Recall = 99.56 F1 Score = 99.45 **Dataset II** Accuracy = 99.97 **Meningioma** Precision = 100 Recall = 100 F1 Score = 100

## Data Availability

In this paper, the dataset is publicly available.
